# Supplementary material for: A quantitative workflow for modeling diversification in material culture
Source: PLoS One. 2020 Feb 6;15(2):e0227579. doi: 10.1371/journal.pone.0227579 (PMC7004301; doi:10.1371/journal.pone.0227579)

Supplemental Information for A Quantiative Workflow for Modeling Diversificatin in Material Culture

Erik Gjesfjeld, Daniele Silvestro, Jonathan Chang, Bernard Koch, Jacob G. Foster, Michael E. Alfaro

January 3, 2020

Introduction

This supplemental information coincides with the preparation of data for the Plos One article titled *A quantitative workflow for modeling diversification in material culture* by Erik Gjesfjeld, Daniele Silvestro, Jonathan Chang, Bernard Koch, Jacob G. Foster and Michael E. Alfaro. As highlighted in the article, we demonstrate the analysis of two main data formats, range format (American automobiles) and occurrence format (radiocarbon dates). The automobile data from America and Europe represents data in the range format as it consists of only the first and last years of production. The radiocarbon data associated with the EuroEvol project represent occurrence format data as it consists of a series of radiocarbon dates assigned to different cultural phrases and periods.

The following supplemental information presents all of the necessary data and code in order to replicate the analysis as presented in the Plos One article, including figures. If any code is not working, please email Erik Gjesfjeld (erik.gjesfjeld@gmail.com).

Loading in necessary libraries and programs

```
library(tidyverse)
library(rcarbon)
library(knitr)
```

In order to start it is necessary to download the most recent versions of PyRate and LiteRate. These are available on the GitHub page of Daniele Silvestro, along with a series of tutorials and example data sets.

- PyRate: https://github.com/dsilvestro/PyRate
- LiteRate: https://github.com/dsilvestro/LiteRate

Preparation of range data

We will start with the analysis of range data and will read in data on automobile production years. The data originally comes from the master vehicle list associated with the Ebay parts and motors database (https://pages.ebay.com/motors/compatibility/download.html). This list records a variety of information in order to list automotive parts for sale. For this research, we will use the fields for make, model, year and region. In total, the database contains 288,056 total entries from the years 1896-2019, updated as of July 2019.

The data was further cleaned to identify car models that had major lapses in production (over 3 years). For these models, a additional "G2" or "G3" was added to identify them as further generation car models. In addition, the nation and continent of the manufacturers headquarters were also added to the dataset.

```r
#Reading in a manually cleaned version of the Ebay Master Vehicle List

download.file("https://ndownloader.figshare.com/files/17608979",
              "~/Desktop/cars_mvl_rev.csv")
mvl_rev <- read.csv("~/Desktop/cars_mvl_rev.csv")
```

American Automobile Data Cleaning and Organization

This dataset also includes commercial trucks that we decided should not be included in the analysis, so this are filtered out based a manually developed list of commercial truck manufacturers (please contact authors for list of commercial trucks).

The following code groups and arranges the data by car model, summarizes the first and last years of production (assuming continual production), filters only American manufacturers and then filters out the trucks by manufacturer, using the list of commercial truck manufacturers that was manually specified above.

```r
#Removing commerical truck manufacuters
cars_mvl <- mvl_rev %>% filter(!make %in% trucks) %>% droplevels()

#Filtering just American manufacturers
cars_mvl_america <- cars_mvl %>% filter(continent=="America")

#Grouping by make / model and taking the first and last years of production (range data)
cars_mvl_america_range<- cars_mvl_america %>% group_by(make_model_rev) %>%
  arrange(year) %>%
  summarise(make=first(make),
            model=first(model),
            first_year=first(year),
            last_year=last(year))

#At the moment of writing, 2018 is the last full calendar year, so we are only using cars
#that originated before 2018, which is considered our time at present.
cars_2018_mvl_america <- cars_mvl_america_range %>% filter(first_year<=2018)
cars_2018_mvl_america$last_year[cars_2018_mvl_america$last_year==2019] <- 2018
cars_2018_mvl_america$last_year[cars_2018_mvl_america$last_year==2020] <- 2018
```

After wrangling the data, it is now ready to be put into range format that will be analysed by LiteRate (see manuscript for details). The default date format for LiteRate is calendar dates (BC / AD), which is what the original data was in and therefore will be maintained.

The fields used will be clade, which are all classified as 1. If you wanted to include additional information such as region of manufacturer it is possible to recode the clade variable to reflect this. Species is simply a unique numeric identifier for each car model in our data. TS refers to the date of origination (time of speciation) and TE refers to the date of last production (time of extinction) or the most recent year of production for cars still being produced (2018 in this study).

```r
cars_2018_pyrate_mvl_america <- data.frame(clade=1, #As we are only using US cars
                              species=as.numeric(as.factor
                                                 (cars_2018_mvl_america$make_model_rev)),
                              ts=cars_2018_mvl_america$first_year, #origination year
                              te=cars_2018_mvl_america$last_year) #extinction year

head(cars_2018_pyrate_mvl_america)
```

```
##   clade species   ts   te
```

```
## 1      1      81 1952 1953
## 2      1      82 1952 1953
## 3      1      95 1958 1974
## 4      1      96 1958 1968
## 5      1      97 1968 1970
## 6      1      98 1978 1980
```

```
write_delim(cars_2018_pyrate_mvl_america,"~/Desktop/cars_2018_mvl_american.txt")
```

Analysis of Automobile Data

The analysis of diversification rates as performed in the manuscript can then be performed by opening a terminal window, navigating to the folder with LiteRateForward.py and executing the following command. The MCMC is run for 20 million (as opposed to the 10 million default) in order to achieve greater convergence.

```
python LiteRateForward.py -d cars_2018_mvl_american.txt -model_BDI 0 -n 20000000 -s 5000
```

Preparation of Occurrence Data

The occurrence data used in the research comes in the form of radiocarbon dates that were collected through the EuroEvol project. The dates and supporting information are openly available from the University College London Discovery repository (http://discovery.ucl.ac.uk/1469811/) with additional details published in Manning et al. 2016 (DOI: http://dx.doi.org/10.5334/joad.40).

```
#Downloading EUROEVOL files from FigShare repository
download.file("https://ndownloader.figshare.com/files/17608922",
              "~/Desktop/common_phases.csv")
ee_phases <- read.csv("~/Desktop/common_phases.csv")

download.file("https://ndownloader.figshare.com/files/17608880",
              "~/Desktop/C14Samples.csv")
ee_c14 <- read.csv("~/Desktop/C14Samples.csv")

#Joining the phase and C14 datasets together by their unique phase code
ee_all<-left_join(ee_c14,ee_phases,by="PhaseCode")
```

Isolating Key Cultures

As highlighted in Manning et al. 2014 (DOI: http://dx.doi.org/10.1017/S0003598X00115327), only 22 of the cultures provided in the dataset have sufficient amounts of data for statistical analysis. Based on this assessment, we are also restricting our analysis to those same 22 cultures.

```
top_22_cultures<- c("Bell Beaker","Cardial","Chasseen","Corded Ware","Cortaillod",
                    "Ertebolle", "Globular Amphora Culture","Horgen","Iwienska","Lengyel",
                    "Linearbandkeramik","Michelsberg","Neolithique final",
                    "Neolithique moyen II","Peu Richard","Pfyn","Pitted Ware","Rossen",
                    "Seine Oise Marne","Trichterbecher","Veraza","Wartberg")
```

As we are only interested in the occurrence data, we remove the other fields in the data.

```
#Filtering out samples with missing data, selecting fields of interest for
#only the 22 cultures with sufficient data
ee_culture_top <- ee_all %>% na.omit()  %>%
```

```r
  filter(!is.na(Culture)) %>% filter(Culture != "NULL") %>%
  select(c("Culture","Period.x","C14Age","C14SD")) %>%
  filter(Culture %in% top_22_cultures) %>% droplevels()  %>% filter(C14Age > 4500)

head(ee_culture_top)
```

```
##              Culture Period.x C14Age C14SD
## 1       Michelsberg       LN   5397    25
## 2           Cardial       EN   6850    65
## 3           Cardial       EN   6745    70
## 4 Linearbandkeramik       EN   6200   190
## 5 Linearbandkeramik       EN   6110   140
## 6 Linearbandkeramik       EN   6030   130
```

```r
#Isolating a vector of the culture names to be used later
culture_names<-as.character(ee_culture_top$Culture)
```

If we want to know how well our filtered data represents different time periods we can create a simple table of the periods designated by the EUROEVOL project.

```r
period_table <- table(ee_culture_top$Period.x, dnn = "Period")
period_code = c("Early Bronze Age",
                "Early/Middle Neolithic",
                "Early Neolithic","Late Mesolithic",
                "Late Mesolithic / Early Neolithic",
                "Late Neolithic",
                "Late Neolithic / Early Bronze Age",
                "Middle/Late Neolithic",
                "Middle Neolithic",
                "Unassigned",
                "Undetermined Neolithic")
row.names(period_table) <- period_code
kable(period_table)
```

Period	Freq
Early Bronze Age	3
Early/Middle Neolithic	29
Early Neolithic	865
Late Mesolithic	377
Late Mesolithic / Early Neolithic	12
Late Neolithic	378
Late Neolithic / Early Bronze Age	0
Middle/Late Neolithic	2
Middle Neolithic	733
Unassigned	226
Undetermined Neolithic	1

Table 1. Frequency of periods represented by the 2,626 radiocarbon dates / 22 cultures used in this analysis.

Calibration of Radiocarbon Dates

As the data are uncalibrated radiocarbon dates, it is necessary to calibrate the dates for them to be meaningful calendar dates. Here, we use the `rcarbon` (Bevan and Crema 2018) package in R for calibration of the 4,243

dates. One of the unique qualities of calibrated radiocarbon dates is the unique probability distribution for each date, which occurs when reflecting the Gaussian distribution of radiocarbon ages against the "wiggles" of the International Calibration curve (intcal13), see image below.

```
#Calibrating the radiocarbon dates (may take between 3-5 minutes)
cal_dates_top<-calibrate(x=ee_culture_top$C14Age,
                         errors = ee_culture_top$C14SD,
                         calCurves = 'intcal13',
                         calMatrix = TRUE)

#Isolating a matrix of the probability distribution from the calibration
cal_dates_mat<-cal_dates_top$calmatrix
```

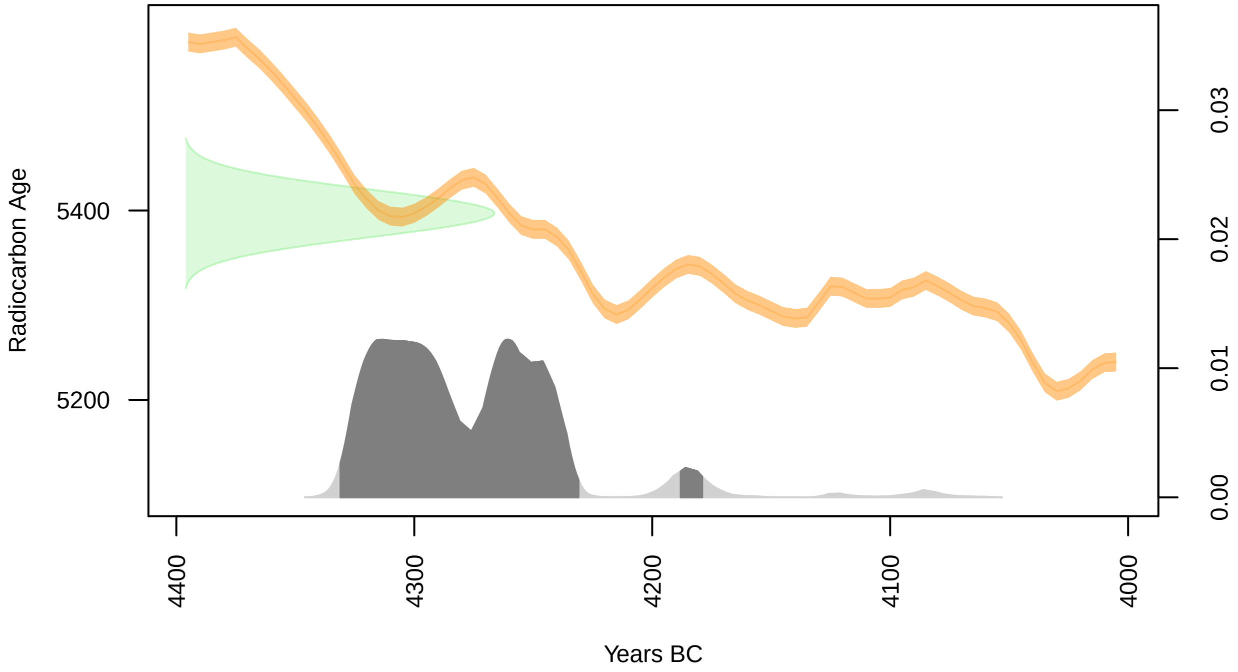

Sampling of Calibrated Radiocarbon Distribution

Due to the unique probability distribution for each radiocarbon date, we randomly sample from the probability distribution for each radiocarbon age. For each single radiocarbon date, we take 100 random samples at yearly intervals contingent on the probabilities of each year based on the probability distribution obtained after calibration. This information is then placed in a table in which ages can be extracted and evaluated in PyRate (see manuscript for additional details).

```
#Creating the function that will sample that probability distribution for
#each radiocarbon date
sample_calib_dates<-function(x,size,sample_names){
sample_df<-matrix(ncol=(size+2),nrow=ncol(x))
sample_df[,1]<-sample_names
sample_df[,2]<-"Extinct"
for (i in 1:ncol(x)){
  pd_samp=sample(x=as.integer(rownames(x)),size=size,replace=TRUE,prob=x[,i])
  sample_df[i,3:(size+2)]<-pd_samp
}
sample_res<-as.data.frame(sample_df)
```

```
colnames(sample_res)<-c("Taxon_name", "Status",
                        paste0("Draw","_",as.character(seq(from=1,to=size,by=1))))
return(sample_res)
}

#Running the function, with 100 samples for each radiocarbon date
#(may take between 5-10 minutes)
sample_res<-sample_calib_dates(cal_dates_mat,100,culture_names)

#Writing text file for extract.ages function in PyRate
write.table(sample_res,"~/Desktop/sample_results_calib.txt",
            quote=FALSE,row.names = FALSE,sep="\t")
```

Extracting Ages

Once the text file of the sampled ages is created, we can use the tools associated with the PyRate program (details of PyRate can be found in the manuscript and associated citations). Here, we will use the pyrate_utilities function in R to extract the ages from our sampled C14 data and format it as occurrence data for analysis in PyRate. Results from this will be output as a .py file.

```
#Sourcing the necessary functions
source("~/path_to_PyRate_folder/pyrate_utilities.r")

#Calling the function to extract C14 ages
extract.ages.14C("~/Desktop/sample_results_calib.txt")
```

Once we have the data in the proper format, this is the file `sample_results_calib_PyRate.py`, we can use PyRate to produce estimates of origination, extinction and preservation rates.

Testing Between Preservation Models

Currently, PyRate has three different models of preservation rates including a Homogeneous Poisson Process (HPP), a non-Homogeneous Poisson Process (NHPP) and a Time-variable Poisson Process (TPP). Here, we will do a simple model test to determine which model best fits our data. As a note, the TPP requires us to construct a set of non-overlapping temporal periods in which preservation rates will be independently calculated for each temporal period. Here, we use a very broad classification scheme of Early (8000-6000 cal BP), Middle (6000-5500 cal BP) and Late Neolithic (5500-3800 cal BP). Of course, dates for these periods are different across regions of Europe, and should therefore be adjusted depending on the region of interest.

```
neo_periods<-c(5500,6000,8000)
write.table(neo_periods,"~/Desktop/neo_periods.txt",
            row.names = FALSE, col.names = FALSE, quote = FALSE)
```

The model test is performed using the PyRate program and is executed in a terminal window using the code below. The flag -qShift refers to the input file of temporal ages for neolithic periods created above. The rescale flag is adjusted for the long time scale and divides each value by 1000 (this creates better convergence of results but does not change the estimates of origination and extinction rates)

```
#These commands should be executed through the Terminal.app
cd ~/Desktop/
python PyRate.py sample_results_calib_PyRate.py -PPmodeltest -qShift neo_periods.txt
-rescale 0.001
```

We can see from the results of this model test the best fitting model is the non-homogeneous Poisson Process (NHPP). Based on these results, we can now execute the full PyRate analysis in a terminal window.

Model	Maximum Likelihood	Preservation Rate(s)	AICc	dAICc
NHPP	-3931.17	79.3	7864	-
TPP	-5180.35	105.05, 101.48,75.30	10368	2503***
HPP	-5235.99	87.53	10474	2509***

PyRate Analysis of Neolithic Europe Radiocarbon Dates

Note: A full PyRate analysis of this occurrence data will likely take anywhere between a few hours on a cluster or a few days on personal laptop or desktop computer. We strongly recommend this command be executed using a computing cluster with remote capabilities.

```
python PyRate.py sample_results_calib_PyRate.py -A 4 -rescale 0.001
-mG -n 10000000 -s 1000 -pP 1 0.01
# -A 4 this uses the RJMCMC algorithm (recommended)
# -mG this uses a gamma preservation model (as is recommended for the NHPP)
# -n this is the total number of samples (default is 10 million)
# -s this is the sampling frequence (default is every 1000 samples)
# -pP adjusts the shape and rate of the preservation prior
```

We will also want to replicate this analysis many times in order to gain confidence in our results and have a robust understanding of the uncertainty around our parameter estimates. Similar to before, we ran 100 replicates `-j ::: {1:100}` using 10 `-j 10` cores of a 16 core virtual machine using a bash shell script and the program parallel. This analysis took 32 hours to complete.

```
parallel --joblog ~/pyrate_replicates_joblog_edge --eta -j 10
python PyRate.py sample_results_calib_PyRate.py -A 4 -n 10000000
-s 1000 -rescale 0.001 -pP 1 0.01 -mG -j ::: {1..100} ##adjusted
```

Once this is finished, we can combine all of the runs into a single combined mcmc file.

```
python PyRate.py -combLogRJ ~/path_to_log_files -b 100
```

With that, all of the analysis has been completed and we are able to visualize the results using the following commands executed in a bash shell.

```
python plotRJForward.py ~/path_to_log_files
python PyRate.py -plotRJ ~/path_to_log_files -tag combined
python PyRate.py -ginput ~/path_to_combined_mcmc.log
```

Creating Figures from the Plos One Article

All the necessary files to recreate the figures produced in the article can be downloaded from the Figshare repository for the article, located here.

In order to recreate the figures, you must download the folder titled 'Files for Figures' to your desktop and execute the commands below. The folder will be titled '11117120' and must be unzipped prior to use.

Figure 1

```
library(tidyverse)
library(scales)

#Figure 1A - Cars Lifespans
cars_2018_American_AD<-read.table("~/Desktop/11117120/cars_2018_mvl_american.txt",
                                  header=TRUE)
cars_2018_america_draw<-arrange(cars_2018_American_AD,ts)
cars_2018_america_draw$ID<-as.numeric(row.names(cars_2018_america_draw))

draw_func_range<-function(x,point_color,point_shape,point_size,line_color,line_width){
  for (i in 1:nrow(x)){
    points(x[i,3],x[i,5],pch=point_shape,cex=point_size,col=as.character(point_color))
    points(x[i,4],x[i,5],pch=point_shape,cex=point_size,col=as.character(point_color))
    segments(x[i,3],x[i,5],x[i,4],x[i,5],col=alpha(as.character(line_color),0.7),
             lwd=line_width,lty=1)
  }
}

par(mar=c(5.1,1.1,1.1,1.1))
plot(cars_2018_america_draw$first_year,
     cars_2018_america_draw$make,type="n",
     ylim=c(0,nrow(cars_2018_america_draw)),
     xlim=c(min(cars_2018_america_draw$ts),max(cars_2018_america_draw$te)),
     yaxt="n",xlab="",ylab="",col.lab="black", col.main="black",bty="n",xaxt="n")
draw_func_range(cars_2018_america_draw,"springgreen4",20,1,"springgreen4",2)
axis(1,at=c(1900,1920,1940,1960,1980,2000,2020),
     labels=c("1900","1920","1940","1960","1980","2000","2020"),cex.axis=1.5)
mtext("Year (AD)", side = 1, line = 3, cex=1.5 )
```

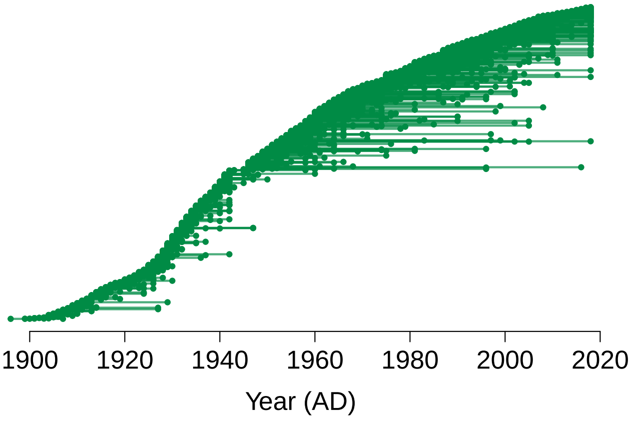


```
#dev.off()

#Figure 1B - Culture Occurrences
samples_draw<-read.delim("~/Desktop/11117120/sample_results_calib.txt")
samples_draw$ID<-as.numeric(as.factor(samples_draw$Taxon_name))
samples_draw<-arrange(samples_draw,ID)
samples_draw_key <- samples_draw %>% group_by(Taxon_name) %>% summarise(ID=first(ID))

par(mar=c(5.1,1.1,1.1,1.1))
```

```
plot(x=samples_draw$Draw_1,y=samples_draw$ID,type="n",ylab="",
     yaxt="n",xlim=c(8000,3000),bty="n",xaxt="n",xlab="")
points(samples_draw$Draw_1,samples_draw$ID,cex=1,pch=20,col="tan4")
axis(1,at=c(8000,7000,6000,5000,4000,3000),
     labels=c("8000","7000","6000","5000","4000","3000"),cex.axis=1.5)
mtext("Year (cal BP)", side = 1, line = 3, cex=1.5 )
```

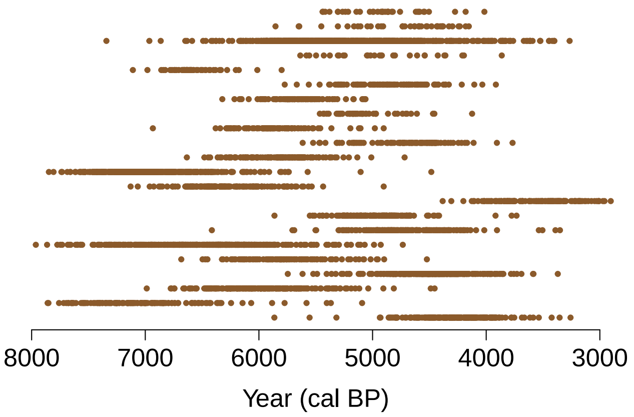

Figure 2

Change over time in the the richness of American car models (A) and archaeological cultures (B) as well as histograms showing the distribution of lifespans for American car models (C) and archaeological cultures (D). The car models with the greatest longevity include the Chrysler Town & Country (1946-2016), the Chevrolet Corvette (1953-2018) and the Cadillac DeVille (1953-2005). The archaeological cultures with the longest lifespans are Trichterbecher, Linearbandkeramik and Ertebolle.

```
library(tidyverse)
library(scales)

cars_2018_American_AD<-read.table("~/Desktop/11117120/cars_2018_mvl_american.txt",
                                  header=TRUE)
cars_2018_American_AD_hist<-cars_2018_American_AD %>% mutate(lifespan=te-ts)
cars_2018_American_AD_counts<-cars_2018_American_AD_hist %>% group_by(lifespan) %>%
  summarise(counts=n())
cars_2018_america_draw<-arrange(cars_2018_American_AD,ts)
cars_2018_america_draw$ID<-as.numeric(row.names(cars_2018_america_draw))

source("~/Desktop/11117120/LTT_data.r")
LTT_data<-source_LTT_data("~/Desktop/11117120/combined_100_mcmc_LTT.r")
occur_richness<-data.frame(time=LTT_data$time,richness=LTT_data$richness,
                           log_rich=log10(LTT_data$richness))

#Figure 2A - Cars Raw Diversity
orig_counts<-cars_2018_america_draw %>% group_by(ts) %>%
  summarize(ts_counts=n()) %>% mutate(ts_sum=cumsum(ts_counts),year=ts)
ex_counts <- cars_2018_america_draw %>% group_by(te) %>%
  summarize(te_counts=n()) %>% mutate(te_sum=cumsum(te_counts),year=te)
raw_diversity <- left_join(orig_counts, ex_counts, by="year") %>%
  mutate(richness=ts_sum-te_sum) %>% mutate(log_rich=log10(richness))

par(mar=c(4.1,4.1,0,1.1))
```

```r
plot(raw_diversity$year[-nrow(raw_diversity)],
     raw_diversity$richness[-nrow(raw_diversity)],
     type="l",bty="n",ylab="",xlab="",col="springgreen4",lwd=3,xaxt="n",yaxt="n")
axis(1,at=c(1900,1920,1940,1960,1980,2000,2020),
     labels=c("1900","1920","1940","1960","1980","2000","2020"),cex.axis=1)
axis(2,at=c(0,50,100,150,200),labels=c("0","50","100","150","200"),cex.axis=1,las=2)
mtext("Year (AD)", side = 1, line = 2, cex=1)
mtext("Richness", side = 2, line = 3, cex=1)
```

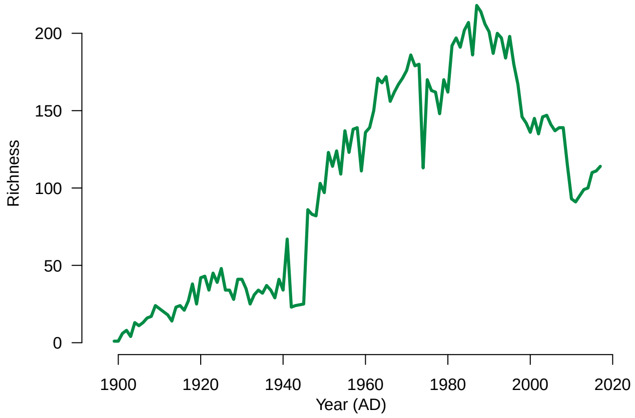


```r
#Figure 2B - Culture Richness
par(mar=c(5.1,2.1,0,1.1))
plot(x=occur_richness$time,y=occur_richness$richness,type="l",xlim=c(7.5,4.5),
     bty="n",yaxt="n",col="tan4",lwd=3,xaxt="n",xlab="",ylab="",cex=1)
axis(1,at=c(7.5,6.5,5.5,4.5),labels=c("7500","6500","5500","4500"),cex.axis=1)
axis(2,at=c(0,5,10,15,20),labels=c("0","5","10","15","20"),cex.axis=1,las=2)
mtext("Year (cal BP)", side = 1, line = 2, cex=1)
```

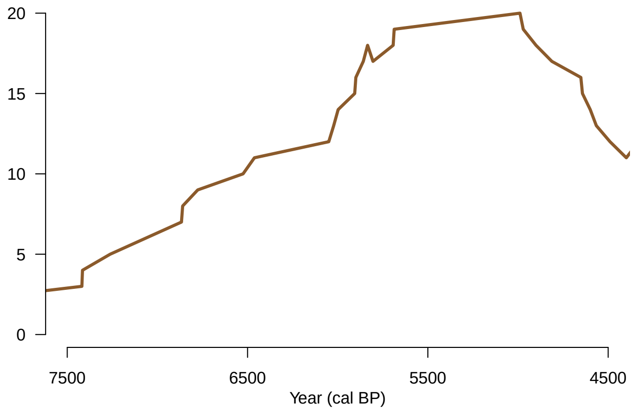


```r
#Figure 2C - Cars Lifespans
par(mar=c(4.1,4.1,1.1,1.1))
plot(y=log10(cars_2018_American_AD_counts$counts),x=cars_2018_American_AD_counts$lifespan,
     type="h",yaxt="n",col="springgreen4",lwd=3,bty="n",ylab="",xlab="",xaxt="n")
axis(1,at=seq(0,70,10),labels=c("0","10","20","30","40","50","60","70"),cex.axis=1)
axis(2,at=c(0,1,2,3),labels=c("0","10","100","1000"),las=2,cex.axis=1)
mtext("Lifespans of Car Models", side = 1, line = 2, cex=1)
mtext("Frequency", side = 2, line = 3, cex=1)
```

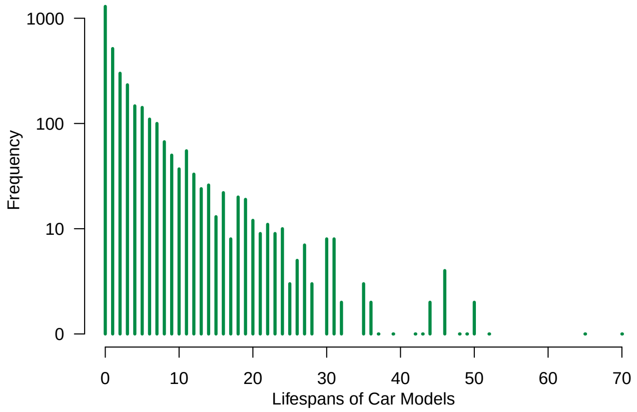


```r
#Figure 2D - Culture Lifespan Histogram
ages<-read.csv("~/Desktop/11117120/sample_results_calib.csv",header=T)
t_max<-ages %>% group_by(Culture) %>% summarise_all(max)
t_max_means <- data.frame(Culture=t_max$Culture
                          ,Means=round(rowMeans(t_max[,2:101]),digits=-2))
t_min<-ages %>% group_by(Culture) %>% summarise_all(min)
t_min_means <- data.frame(Culture=t_min$Culture,
                          Means=round(rowMeans(t_min[,2:101]),digits=-2))
t_life <- data.frame(Culture=t_min$Culture,ts=t_max_means$Means,
                     te=t_min_means$Means) %>% mutate(lifespan=ts-te)
occur_counts <- t_life %>% group_by(lifespan) %>% summarise(counts=n())

par(mar=c(5.1,2.1,1.1,1.1))
plot(y=occur_counts$counts,x=occur_counts$lifespan,type="h",col="tan4",
     lwd=3,bty="n",ylab="",xlab="",xaxt="n",xlim=c(1000,3000),ylim=c(0,6),yaxt='n')
axis(1,at=c(1000,2000,3000),labels=c("1000","2000","3000"),cex.axis=1)
axis(2,at=seq(0,5,1),labels=c("0","1","2","3","4","5"),cex.axis=1,las=2)
mtext("Lifespans of Cultures", side = 1, line = 2, cex=1 )
```

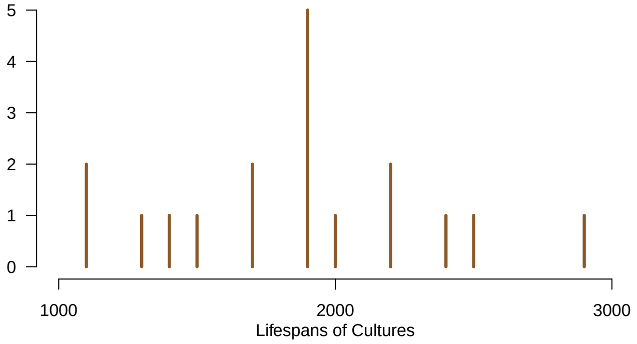

Figure 3

Rates of diversification through time for American car models. A) Solid lines indicate mean origination and extinction rate for each year with shaded areas representing 95% highest probability densities. B) Changes in the net diversification rate through time (origination rate – extinction rate) with the dotted line indicated net diversification of 0. C) Frequency of rate shifts based on posterior samples for each time unit of our analysis. Dotted lines indicate the strength of evidence for the rate shift frequency as corresponding to the standard natural log Bayes Factors thresholds of 2 (lower dotted line) and 6 (higher dotted line).

```r
library(tidyverse)
library(scales)

#Load Function for Getting the Data
source("~/Desktop/11117120/RTT_data_literate.R")
source("~/Desktop/11117120/Shift_data_literate.R")
plot_cars<-source_RTT_data("~/Desktop/11117120/RTT_plots_cars.r")
plot_cars_shifts<-source_Shift_data("~/Desktop/11117120/RTT_plots_cars.r")

sp_top_shifts<-which(plot_cars_shifts$sp_counts>bf6)-1
ex_top_shifts<-which(plot_cars_shifts$ex_counts>bf6)-1

#Figure 3A - Origination / Extinction Rates
par(mar=c(5.1,3.1,0.2,1.5))
plot(plot_cars$time,plot_cars$time,type = 'n', ylim = c(0, 0.9781390361293459),
     xlim = c(1896.0,2018.5), ylab = '', xlab = '',bty="n",xaxt="n",yaxt="n")
axis(1,at=seq(1900,2020,by=20),
     labels=c("1900","1920","1940","1960","1980","2000","2020"),cex.axis=1.5)
axis(2,at=seq(0,1,by=0.2),labels=c("0","0.2","0.4","0.6","0.8","1"),
     cex.axis=1.5,las=2)
mtext("Year (AD)", line = 3, side =1, cex = 1.5)

polygon(c(plot_cars$time, rev(plot_cars$time)), c(plot_cars$sp_max_HPD,
                                                   rev(plot_cars$sp_min_HPD)),
        col = alpha('navyblue',0.3), border = NA)
lines(plot_cars$time,plot_cars$sp_rate, col = 'navyblue', lwd=2)

polygon(c(plot_cars$time, rev(plot_cars$time)), c(plot_cars$ex_max_HPD,
                                                   rev(plot_cars$ex_min_HPD)),
        col = alpha('firebrick',0.3), border = NA)
lines(plot_cars$time,plot_cars$ex_rate, col = 'firebrick', lwd=2)

points(plot_cars[sp_top_shifts,1],plot_cars[sp_top_shifts,2],
       pch=16,col="navyblue",cex=1.5)
points(plot_cars[ex_top_shifts,1],plot_cars[ex_top_shifts,3],
       pch=18,col="firebrick",cex=1.5)

rect(1960,0.80,1970,0.88,col=alpha("firebrick",0.3),border=NA)
segments(1960,0.84,1970,0.84,col="firebrick",lwd=2)
rect(1960,0.91,1970,0.99,col=alpha("navyblue",0.3),border=NA)
segments(1960,0.95,1970,0.95,col="navyblue",lwd=2)

points(1960,0.70,pch=16,col="navyblue",cex=1.5)
points(1960,0.60,pch=18,col="firebrick",cex=1.5)

text("Origination Rate", pos=4, x=1970,y=0.95,cex=1.5)
text("Extinction Rate", pos=4, x=1970,y=0.84,cex=1.5)
text("Origination Shift Points",pos=4,x=1960,y=0.70,cex=1.5)
text("Extinction Shift Points",pos=4,x=1960,y=0.60,cex=1.5)
```

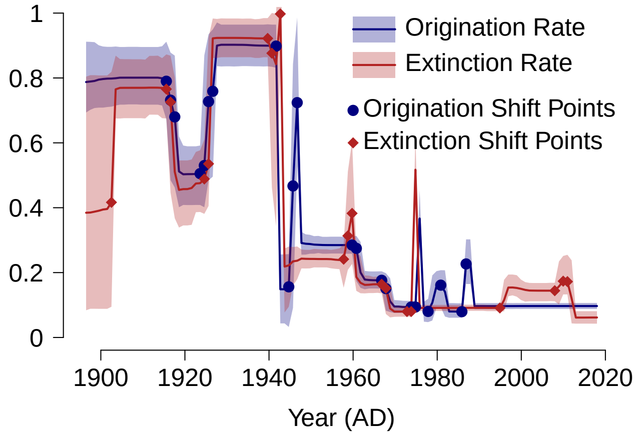


```
#Figure 3B - Net Diversification Plot
par(mar=c(5.1,3.1,0.2,1.5))
plot(plot_cars$time,plot_cars$time,type = 'n',
     ylim = c(-0.860041756138627, 0.840821109350957 5),
     xlim = c(1896.0,2018.5), ylab = '', xlab = '',lwd=2, main='', col= 'purple4' ,
     bty="n",xaxt="n",yaxt="n")
axis(1,at=seq(1900,2020,by=20),
     labels=c("1900","1920","1940","1960","1980","2000","2020"),cex.axis=1.5)
axis(2,at=seq(-0.8,0.8,by=0.4),
     labels=c("-0.8","-0.4","0.0","0.4","0.8"),cex.axis=1.5,las=2)
mtext("Year (AD)", line = 3, side =1, cex = 1.5)

polygon(c(plot_cars$time, rev(plot_cars$time)), c(plot_cars$net_max_HPD,
                                                   rev(plot_cars$net_min_HPD)),
        col = alpha('purple4',0.3), border = NA)
lines(plot_cars$time,plot_cars$net_rate, col = 'purple4', lwd=2)
abline(h=0,lty=2)

rect(1955,0.51,1965,0.59,col=alpha("purple4",0.3),border=NA)
segments(1955,0.55,1965,0.55,col="purple4",lwd=2)
text("Net Diversification Rate", pos=4, x=1965,y=0.55,cex=1.5)
```

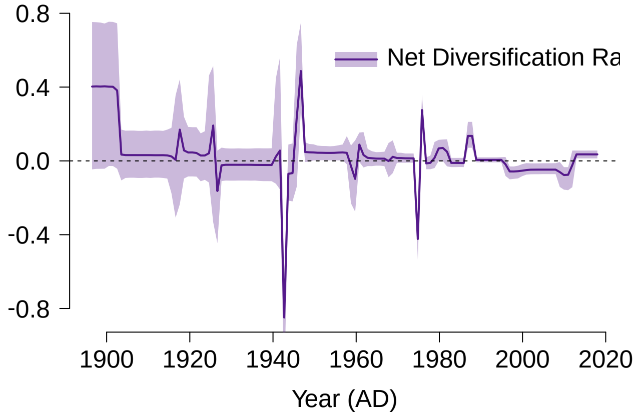


```
#Figure 3C - Origination Frequency Shifts
par(mar=c(4.1,4.1,0.2,1.1))
plot(plot_cars_shifts$mids,plot_cars_shifts$sp_counts,type = 'n', bty="n",
     xlim = c(1896,2018.5),
     ylab = '', xlab ='',lwd=7,col=alpha('firebrick',0.5),
```

```
     ylim=c(0,1),xaxt="n",yaxt="n")
lines(plot_cars_shifts$mids,plot_cars_shifts$sp_counts,type = 'h',
      ylab = '', xlab = '',lwd=3,col=alpha('navyblue',0.8))
axis(1,at=seq(1900,2020,by=20),
     labels=c("1900","1920","1940","1960","1980","2000","2020"),cex.axis=1.5)
axis(2,at=seq(0,1,by=0.2),
     labels=c("0","0.2","0.4","0.6","0.8","1"),cex.axis=1.5,las=2)
mtext("Frequency of Rate Shift", line=2.85, side=2, cex=1.5)
mtext("Year (AD)", line = 2.5, side =1, cex = 1.5)
abline(h=bf2,lty=2)
abline(h=bf6,lty=2)
points(plot_cars_shifts[sp_top_shifts+1,1],
       plot_cars_shifts[sp_top_shifts+1,2],pch=20,col="navyblue",cex=1.5)
text("Origination",x=1915,y=0.95,cex = 1)
```

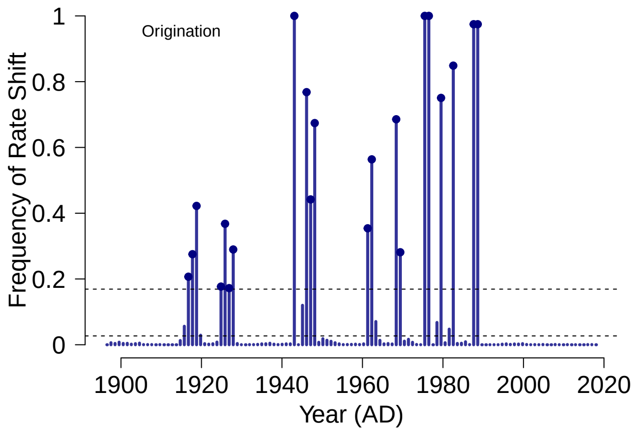


```
#Figure 3D - Extinction Frequency Shifts
par(mar=c(4.1,0.2,0.2,1.1))
plot(plot_cars_shifts$mids,plot_cars_shifts$ex_counts,
     type = 'n', bty="n",xlim = c(1896,2018.5),
     ylab = '', xlab ='',lwd=7,col=alpha('firebrick',0.5),
     ylim=c(0,1),xaxt="n",yaxt="n")
lines(plot_cars_shifts$mids,plot_cars_shifts$ex_counts,
      type = 'h', ylab = '', xlab = '',lwd=3,col=alpha('firebrick',0.8))
axis(1,at=seq(1900,2020,by=20),
     labels=c("1900","1920","1940","1960","1980","2000","2020"),cex.axis=1.5)
mtext("Year (AD)", line = 2.5, side =1, cex = 1.5)
abline(h=bf2,lty=2)
abline(h=bf6,lty=2)
points(plot_cars_shifts[ex_top_shifts+1,1],
       plot_cars_shifts[ex_top_shifts+1,3],pch=18,col="firebrick",cex=1.5)
text("Extinction",x=1915,y=0.95,cex = 1)
```

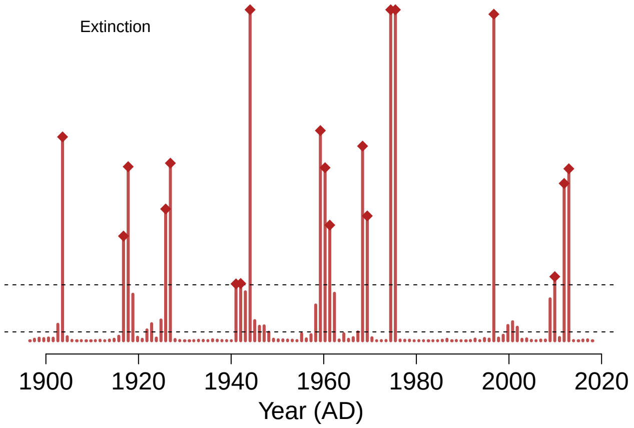

Figure 4

Rates of diversification through time for cultures of the European Neolithic. A) Solid lines indicate mean origination and extinction rate for each year with shaded areas representing 95% highest probability densities. B) Changes in the net diversification rate through time (origination rate – extinction rate) with the dotted line indicated net diversification of 0. C) Frequency of rate shifts based on posterior samples for each time unit of our analysis. Dotted lines indicate the significance of the rate shift frequency as corresponding to the logged Bayes Factors of 2 (lower dotted line) and 6 (higher dotted line).

```
library(tidyverse)
library(scales)

#Load Function for Getting the Data
source("~/Desktop/11117120/RTT_data.R")
source("~/Desktop/11117120/Shift_data.R")

#Load the data after performing the -plotRJ function in shell
plot_cult<-source_RTT_data("~/Desktop/11117120/RTT_plots_neo.r")
plot_cult_shifts<-source_Shift_data("~/Desktop/11117120/RTT_plots_neo.r")

sp_top_shifts <- filter(plot_cult_shifts,sp_counts >bf6)
ex_top_shifts <- filter(plot_cult_shifts,ex_counts>bf6)

#Figure 4A - Origination / Extinction Rates
par(mar=c(5.1,3.1,0.2,1.5))
plot(plot_cult$time,plot_cult$time,type = 'n', ylim = c(0, 2), xlim = c(-7.5,-4.5),
     ylab = '', xlab = '',bty="n",xaxt="n",yaxt="n")
axis(1,at=seq(-7.5,-4.5,by=1),labels=c("7500","6500","5500","4500"),cex.axis=1.5)
axis(2,at=seq(0,2,by=0.5),labels=c("0","0.5","1.0","1.5","2.0"),cex.axis=1.5,las=2)
mtext("Year (cal BP)", line = 3, side =1, cex = 1.5)

polygon(c(plot_cult$time, rev(plot_cult$time)),
        c(plot_cult$sp_max_HPD, rev(plot_cult$sp_min_HPD)),
        col = alpha('navyblue',0.3), border = NA)
lines(plot_cult$time,plot_cult$sp_rate, col = 'navyblue', lwd=2)

polygon(c(plot_cult$time, rev(plot_cult$time)),
        c(plot_cult$ex_max_HPD, rev(plot_cult$ex_min_HPD)),
```

```r
        col = alpha('firebrick',0.3), border = NA)
lines(plot_cult$time,plot_cult$ex_rate, col = 'firebrick', lwd=2)

#These are hardcoded in because the frequency of rate shift counts 100 time bins from
#0 to 8000, whereas the origination and extinction rates count 100 time bins from 3000
#to 8000 (the time range of the actual data). Therefore, I have manually identified the
#time periods that closest approximately the age from the frequency counts for the rates
points(plot_cult[47,1],plot_cult[47,2],pch=16,col="navyblue",cex=1.5)
points(plot_cult[48,1],plot_cult[48,2],pch=16,col="navyblue",cex=1.5)
points(plot_cult[50,1],plot_cult[50,2],pch=16,col="navyblue",cex=1.5)
points(plot_cult[52,1],plot_cult[52,2],pch=16,col="navyblue",cex=1.5)
points(plot_cult[36,1],plot_cult[36,3],pch=18,col="firebrick",cex=1.5)
points(plot_cult[37,1],plot_cult[37,3],pch=18,col="firebrick",cex=1.5)
points(plot_cult[39,1],plot_cult[39,3],pch=18,col="firebrick",cex=1.5)
points(plot_cult[40,1],plot_cult[40,3],pch=18,col="firebrick",cex=1.5)

rect(-7.5,1.8,-7.2,2,col=alpha("navyblue",0.3),border=NA)
segments(-7.5,1.9,-7.2,1.9,col="navyblue",lwd=2)
rect(-7.5,1.5,-7.2,1.7,col=alpha("firebrick",0.3),border=NA)
segments(-7.5,1.6,-7.2,1.6,col="firebrick",lwd=2)

points(-6,1.9,pch=16,col="navyblue",cex=1.5)
points(-6,1.6,pch=18,col="firebrick",cex=1.5)

text("Origination Rate", pos=4, x=-7.2,y=1.9,cex=1.5)
text("Extinction Rate", pos=4, x=-7.2,y=1.6,cex=1.5)
text("Origination Shift Points",pos=4,x=-6,y=1.9,cex=1.5)
text("Extinction Shift Points",pos=4,x=-6,y=1.6,cex=1.5)
```

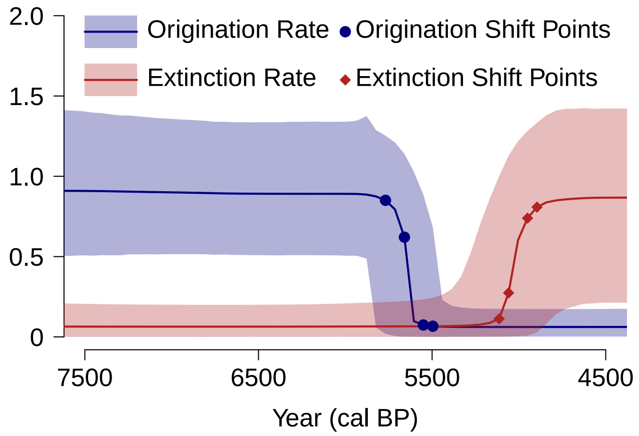


```r
#Figure 2B - Net Diversification Plot
par(mar=c(5.1,3.5,0.2,1.5),xpd=FALSE)
plot(plot_cult$time,plot_cult$time,type = 'n', ylim = c(-1.5,1.5),
     xlim = c(-7.5,-4.5), ylab = '', xlab = '',lwd=2, main='', col= 'purple4' ,bty="n",
     xaxt="n",yaxt="n")
axis(1,at=seq(-7.5,-4.5,by=1),labels=c("7500","6500","5500","4500"),cex.axis=1.5)
axis(2,at=seq(1.5,-1.5,by=-0.5),labels=c("1.5","1.0","0.5","0","-0.5","-1.0","-1.5"),
     cex.axis=1.5,las=2)
mtext("Year (cal BP)", line = 3, side =1, cex = 1.5)

polygon(c(plot_cult$time, rev(plot_cult$time)), c(plot_cult$net_max_HPD,
```

```
                                        rev(plot_cult$net_min_HPD)),
        col = alpha('purple4',0.3), border = NA)
lines(plot_cult$time,plot_cult$net_rate, col = 'purple4', lwd=2)
segments(-7.5,0,-4.5,0,lty=2)

rect(-7.5,-1,-7.2,-0.6,col=alpha("purple4",0.3),border=NA)
segments(-7.5,-0.8,-7.2,-0.8,col="purple4",lwd=2)
text("Net Diversification Rate", pos=4, x=-7.2,y=-0.8,cex=1.5)
```

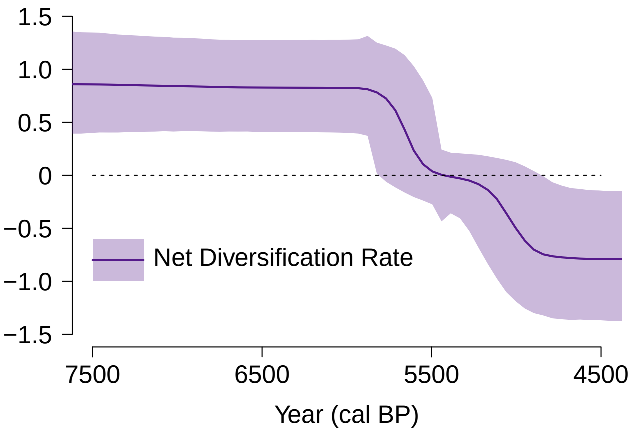


```
#Figure 4C - Origination Frequency Shifts
par(mar=c(4.1,4.1,0.2,0.8))
plot(plot_cult_shifts$mids,plot_cult_shifts$sp_counts,type = 'n', bty="n",
     xlim = c(-7.5,-4.5),ylab = '', xlab = '',lwd=7,col=alpha('navyblue',0.5),
     ylim=c(0,0.4),xaxt="n",yaxt="n")
lines(plot_cult_shifts$mids,plot_cult_shifts$sp_counts,type = 'h', ylab = '',
      xlab = '',lwd=3,col=alpha('navyblue',0.8))
axis(1,at=seq(-7.5,-4.5,by=1),labels=c("7500","6500","5500","4500"),cex.axis=1.5)
axis(2,at=seq(0,0.4,by=0.1),labels=c("0","0.1","0.2","0.3","0.4"),cex.axis=1.5,las=2)
mtext("Frequency of Rate Shift", line=2.85, side=2, cex=1.5)
mtext("Year (cal BP)", line = 2.5, side =1, cex = 1.5)
abline(h=bf2,lty=2)
abline(h=bf6,lty=2)
points(plot_cult_shifts[65:68,1],plot_cult_shifts[65:68,2],pch=20,col="navyblue",cex=1.5)
text("Origination",x=-7,y=0.375,cex = 1)
```

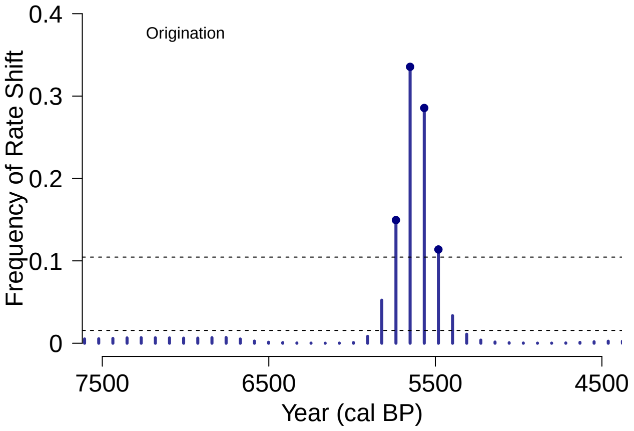

```r
#Figure 4D - Extinction Frequency Shifts
par(mar=c(4.1,1.5,0.2,1.1))
plot(plot_cult_shifts$mids,plot_cult_shifts$ex_counts,type = 'n', bty="n",
     xlim = c(-7.5,-4.5),ylab = '', xlab = '',lwd=7,col=alpha('firebrick',0.5),
     ylim=c(0,0.4),xaxt="n",yaxt="n")
lines(plot_cult_shifts$mids,plot_cult_shifts$ex_counts,type = 'h', ylab = '',
      xlab = '',lwd=3,col=alpha('firebrick',0.8))
axis(1,at=seq(-7.5,-4.5,by=1),labels=c("7500","6500","5500","4500"),cex.axis=1.5)
mtext("Year (cal BP)", line = 2.5, side =1, cex = 1.5)
abline(h=bf2,lty=2)
abline(h=bf6,lty=2)
points(plot_cult_shifts[58:61,1],plot_cult_shifts[58:61,3],pch=18,col="firebrick",cex=1.5)
text("Extinction",x=-7,y=0.375,cex = 1)
```

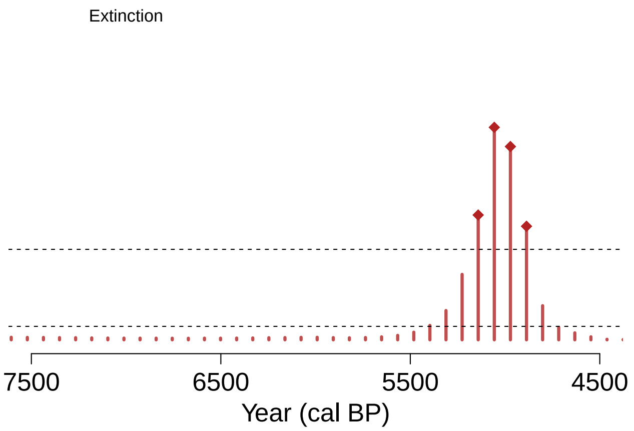

Figure 5

Overall log richness of archaeological cultures (i.e. pottery types) (A) with three scenarios that would produce a similar pattern of rising diversity followed by a decline in diversity. This includes a A) a constant origination rate and increasing extinction rate; B) a declining origination rate and constant extinction rate; and C) a declining origination rate and increasing extinction rate.

```r
x_time <- seq(100,1,-1)
flat<-rep(25,100)
rise<-seq(1,100,1)
B_line <- seq(0,33,by=0.33)
C_line <- seq(50,17,by=-0.33)

source("~/Desktop/11117120/LTT_data.r")
LTT_data<-source_LTT_data("~/Desktop/11117120/combined_100_mcmc_LTT.r")
occur_richness<-data.frame(time=LTT_data$time,richness=LTT_data$richness,
                           log_rich=log10(LTT_data$richness))

par(mar=c(5.1,5.1,1.1,3.1),xpd=FALSE)
plot(plot_cult$time,plot_cult$time,type = 'n', ylim = c(0,1.5), xlim = c(-7.5,-4.5),
     ylab = '', xlab = '',bty="n",xaxt="n",yaxt="n")
axis(1,at=seq(-7.5,-4.5,by=1),labels=c("7500","6500","5500","4500"),cex.axis=1.5)
axis(2,at=seq(0,1.5,by=0.5),labels=c("0","0.5","1.0","1.5"),cex.axis=1.5,las=2)
lines(x=-occur_richness$time[3:35],y=occur_richness$log_rich[3:35],
```

```
      type="l",lwd=4,col="tan4")
mtext("Year (cal BP)", line = 3, side =1, cex = 1.5)
mtext("Richness of Cultures (Log)",line=3, side=2,col="black",cex=1.5)
```

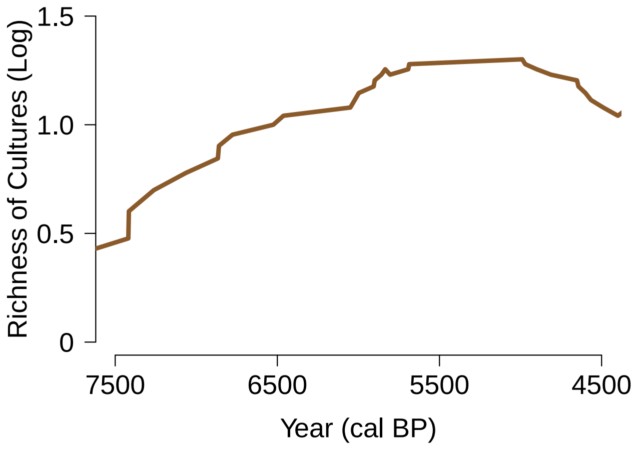


```
plot(x_time,rise,xlim=c(100,1),type='n',xaxt="n",yaxt="n",bty="n",ylab="",
     xlab="",ylim=c(0,50))
arrows(x_time[1],flat[1],x_time[100],flat[100],col="navyblue",lwd=4,length=0.1)
arrows(x_time[1],B_line[1],x_time[100],B_line[100],col="firebrick",lwd=4,length=0.1)
```

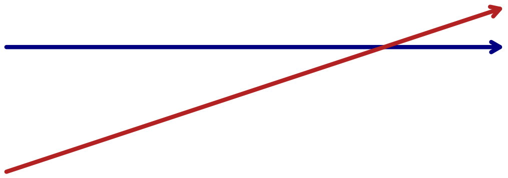

```
plot(x_time,rise,xlim=c(100,1),type='n',xaxt="n",yaxt="n",bty="n",ylab="",
     xlab="",ylim=c(0,50))
arrows(x_time[1],flat[1],x_time[100],flat[100],col="firebrick",lwd=4,length=0.1)
arrows(x_time[1],C_line[1],x_time[100],C_line[100],col="navyblue",lwd=4,length=0.1)
```

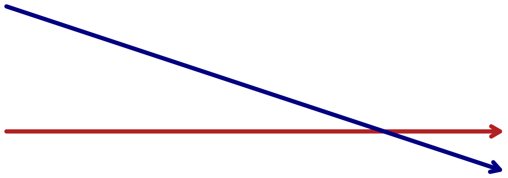

```
plot(x_time,rise,xlim=c(100,1),type='n',xaxt="n",yaxt="n",bty="n",ylab="",
     xlab="",ylim=c(0,50))
arrows(x_time[1],B_line[1]/3,x_time[100],B_line[100]/3,col="firebrick",lwd=4,length=0.1)
arrows(x_time[1],C_line[1]/3,x_time[100],C_line[100]/3,col="navyblue",lwd=4,length=0.1)
```

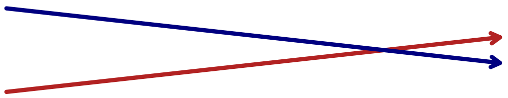

Supplement: S1 Data — (PDF) [file pone.0227579.s001.pdf]
